# Maize Yield Response, Root Distribution and Soil Desiccation Crack Features as Affected by Row Spacing

**DOI:** 10.3390/plants12061380

**Published:** 2023-03-20

**Authors:** Giovanni Lacolla, Davide Caranfa, Ugo De Corato, Giovanna Cucci, Mario Alberto Mastro, Anna Maria Stellacci

**Affiliations:** 1 Department of Soil, Plant and Food Science (Di.S.S.P.A.), University of Bari ‘Aldo Moro’, Via Amendola 165/A, 70126 Bari, Italy; 2 Department of Bioenergy, Biorefinery and Green Chemistry, Italian National Agency for the New Technologies, Energy and Sustainable Economic Development (DTE-BBC-BIC-ENEA), Territorial Office of Bari, 70125 Bari, Italy

**Keywords:** plant density, crack features, crack surface and volume, soil evaporation, in situ mapping of root spatial distribution

## Abstract

Plant density is among the most critical factors affecting plant yields and resource use efficiency since it drives the exploitation of the available resources per unit area, root distribution and soil water losses by direct evaporation from the soil. Consequently, in fine-textured soils, it can also affect the formation and development of desiccation cracks. The aim of this study, carried out on a sandy clay loam soil in a typical Mediterranean environment, was to investigate the effects of different row spacings of maize (*Zea mais* L.) on yield response, root distribution and the main features of desiccation cracks. The field experiment compared bare soil and soil cropped with maize using three plant densities (6, 4 and 3 plants m^−2^), obtained by keeping the number of plants in a row constant and varying the distance between the rows (0.5–0.75–1.0 m). The highest kernel yield (16.57 Mg ha^−1^) was obtained with the greatest planting density (6 plants m^−2^) with a row spacing of 0.5 m; significantly lower yields were recorded with spacings of 0.75 and 1 m, with a decrease of 8.09% and 18.24%, respectively. At the end of the growing season, soil moisture in the bare soil was on average 4% greater in comparison to the cropped soil and was also affected by row spacing, decreasing with the decrease in the inter-row distance. An inverse behaviour was observed between soil moisture and both root density and desiccation crack size. Root density decreased to the increase in soil depth and to the increase in distance from the row. The pluviometric regime occurred during the growing season (total rainfall of 343 mm)-resulted in the formation of cracks of reduced size and with an isotropic behaviour in the bare soil, whereas in the cultivated soil, the cracks were parallel to the maize rows and increased in size with decreasing inter-row distance. The total volume of the soil cracks reached a value of 135.65 m^3^ ha^−1^ in the soil cropped with a row distance of 0.5 m, and was about ten times greater in comparison to the bare soil and three times greater in comparison to a row spacing of 1 m. Such a volume would allow a recharge of 14 mm in the case of intense rainy events on soil characterised by low permeability.

## 1. Introduction

Among the factors that affects maize (*Zea mais* L.), yield response, plant density and row distance play a critical role [1]. Row spacing significantly affects the uniformity of plant distribution and defines the overall plant density. Since plants compete for nutrients, light and other growth factors, equally spaced plants would show minimum competition and thus maximum performance in terms of growth and yield [2] through a better exploitation of the available resources per unit area [3,4]. Previous studies have shown that reduction of row spacing also decreases soil heterogeneity [2]. Although optimal row spacing varies as a function of the crop, maximum yield response will be achieved using equally spaced plants, as this will ensure optimal use of water, nutrients and solar radiation [5]. Therefore, the absence of density stress plays an important role for increasing resource use efficiency and for the improvement of individual plant yield potential [6].

Research on row spacing in maize has provided contrasting results [1,7]. In recent years, narrow row-spaced maize has been suggested as the optimal cropping technique for improving kernel yields [8]. For instance, Porter et al. [9] observed an increase of 7% in grain yield in research performed in Minnesota, while Nielsen [10] in India, reported an increase of 3% in the grain yield for narrow row-spaced maize in comparison to the conventional row-spaced maize (row spacing of 0.76 m). More recently, Widdicombe and Thelen [11] observed that maize grown with narrow row spacing (0.38 and 0.56 m) provided a 4% greater grain yield in comparison to the conventional row-spaced maize (row spacing of 0.76 m) in Michigan. The behaviour described seems to be generally accepted for maize grown in northern locations [12]. However, yield decrements have been also reported in response to narrower row spacings [4]. Pedersen and Lauer [13] found, for instance, a 11% lower yield for maize grown with 0.19 m row spacing in comparison to the crops grown with 0.38 and 0.76 m spacing.

Row spacing affects canopy architecture that in turns affects the utilization of light, water and nutrients. Earlier canopy closure in maize grown in narrower rows has been found to enhance light interception [14] and improve the efficiency of water use in comparison to the crops grown in conventional row spacing (0.81 and 1.07 m) [15]. When the solar radiation moves through the vegetation, a reduction of the photon flux density and a modification of its spectral characteristics occur when the vegetation is dense [16].

Plant density also affects root density and distribution and consequently drives root water uptake. Maize grown in narrow rows generally shows greater root density with a consequent reduction of direct evaporation from the soil and a greater water uptake. Consequently, these hydric relations may result in the formation and development of desiccation cracks in fine-textured soils. Regardless from the top layer water content, water losses by direct evaporation from the soil can be greatly affected by the presence of soil desiccation cracks and their sizes. A crack network may show preferential directions with differences in the average crack dimensions, average depth and distance between cracks. Cropping techniques such as shallow tillage in the inter-row and crop row distance can affect the characteristics of the soil cracks and the overall crack network [17]. Cantore et al. [18] observed that soil cracking caused a contact surface between the soil and the atmosphere that was 4.5 times greater than that recorded in the same soil after soil hoeing. The inner surface of the soil cracks (crack wall surface) may also widely vary as a function of soil texture and soil water content and may reach values between 2.9 and 4.6 times the surface area in clay and dry soils [18]. Overall, the presence of vegetation, especially plant distribution, is highly involved in the development and expansion of desiccation cracks [19] since plant roots tend to cluster in the areas characterized by a lower penetration resistance such as the inter-row area or along the cracks of the swelling clay soils [20,21]. Therefore, the cropping system together with its management can greatly affect the hydric relations in the soil–plant–atmosphere continuum and consequently the water storage capability of the soil, an issue that assumes an ever increasing importance particularly in the view of climate change scenarios. Notwithstanding the crucial role of the factors described in the efficient use of water and natural resources and in crop performance, few studies have focused on the simultaneous assessment of the effect of row spacing on crop response and hydric relations in the soil–plant system and on the consequent formation of desiccation cracks.

For this reason, the aim of this study was to verify how different row spacings could affect the interactions in the soil–plant system continuum by simultaneously investigating plant yield response, root distribution and the main features of soil desiccation cracks. The study was carried out on a sandy clay loam soil in a typical Mediterranean environment; the field experiment compared bare soil and soil cropped with maize (hybrid “P1547, Pioneer”, FAO class 600) using three different plant densities (6, 4 and 3 plants m^−2^) obtained by keeping the number of plants in a row constant and varying the distance between the rows (0.5–0.75–1.0 m).

## 2. Results

**Figure 1:**
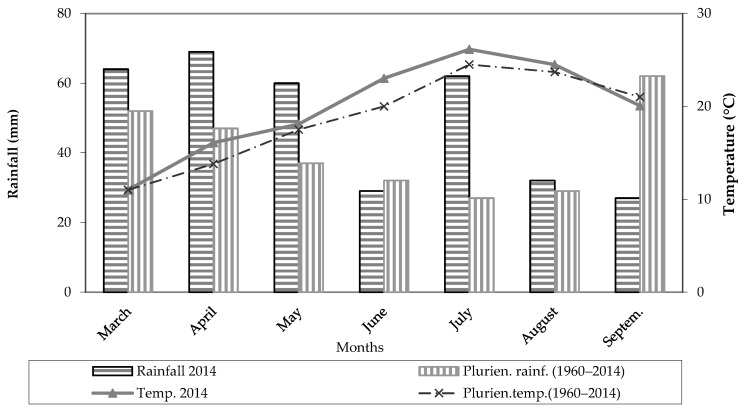
Monthly mean temperatures and total monthly rainfall during the maize cropping cycle for the study year and the long-term period (1960–2014).

The thermo-pluviometric trend observed during the maize cropping cycle is reported in Figure 1. A total amount of rainfall of 343 mm was recorded during the growing season, with rain events quite evenly distributed over the season; monthly values slightly higher than 60 mm were recorded in the period March–May and in July, whereas values of 29, 32 and 27 mm were recorded in the months of June, August and September, respectively (Figure 1). An average temperature of 19.85 °C was recorded over the growing season; in the June to August period, air temperatures reached an average value of 24.56 °C (Figure 1).

### 2.1. Plant Height, Dry Aboveground Biomass, and Plant Nutritional Status

Biometric variables were significantly affected by the treatments. Plant height increased progressively with the decrease in plant row spacing, from values of 2.69 to 2.82 and 2.96 m with spacing decreasing from 1.00 to 0.75 and 0.50 m, respectively (Table 1).

The behaviour observed may be attributed to the greater competition for solar radiation occurring with the narrower row distances that resulted in greater plant heights. Different planting strategy also significantly affected the dry aboveground biomass. The highest average value (29.81 Mg ha^−1^) was observed with the greatest plant density (6 plants m^−2^, with a row spacing of 0.50 m) and decreases of 12.24% and 22.20% were observed with spacings of 0.75 and 1 m, corresponding to plant densities of 4 and 3 plants m^−2^ (Table 1), respectively.

**Figure 2:**
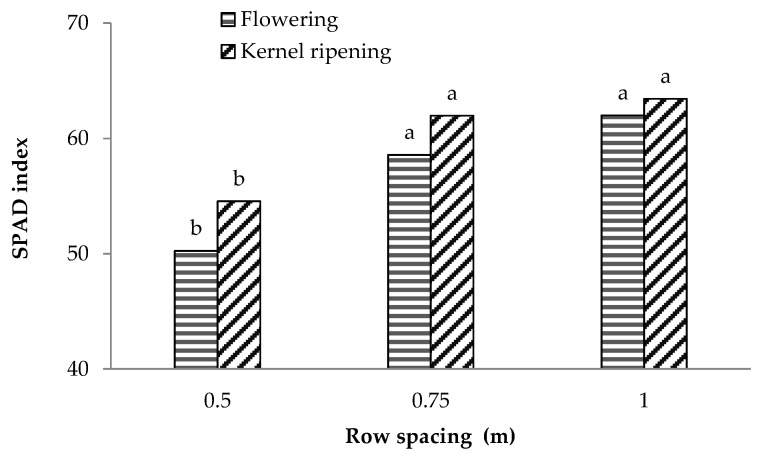
Mean SPAD values observed at flowering and kernel ripening phenological stages in maize cultivar P1547 grown with different row spacings (0.5 m, 0.75 m, 1 m). The bars with the same letter within each phenological stage are not significantly different (SNK test at *p* = 0.05).

An inverse behaviour was observed for the plant nutritional status. The total chlorophyll content, estimated at the flowering and kernel ripening stages using the SPAD index [22,23], was significantly affected by row spacing with the lowest values recorded for the crops grown in narrower rows (0.5 m) at both phenological stages. Larger row spacings (0.75 and 1 m) did not significantly affect the crop nutritional status (Figure 2) and a similar behaviour was observed at both flowering and kernel ripening stages.

### 2.2. Quantitative and Qualitative Maize Yield Response

The kernel yield response, evaluated at a moisture content of 14%, reflected the behaviour shown by the biometric variables (plant height and dry aboveground biomass). The highest kernel yield (16.57 Mg ha^−1^) was recorded with the greatest plant density, while a decrease of 8.09% was observed with a plant density of 4 plants m^−2^. Finally, the lowest yield response, with a decrease of 18.24% in comparison to the highest yield value, was observed for the crop grown with the lowest plant density (3 plants m^−2^ and row spacing of 1 m; Table 1).

For a deeper understanding of the maize yield response to the treatments under study, the thousand seed weight and the hectolitre kernel weight were quantified. Specifically, the first indicator provides information of the kernel size whereas the second represents a qualitative and technological indicator directly related to the milling yield. The highest value of the thousand seed weight (352.07 g) was recorded for the crop grown with the greatest row spacing (1 m) while significantly lower values were observed with narrower distances (Table 1). A similar behaviour was observed for the hectolitre kernel weight that showed the highest value (74.25 kg hL^−1^) for the crops grown with the maximum row spacing (1 m) and a significant decrease with decreasing row spacing (Table 1).

Finally, the harvest index (HI) was not affected by the treatments.

### 2.3. Water Evaporation and Soil Moisture

Both the presence of the crop, in comparison to the bare soil, and the different row spacings significantly affected the hydric relations in the soil–plant system and consequently the root growth and the characteristics of the crack network. Specifically, the different treatments affected the water evaporation from the soil surface and the soil moisture content, as well as the spatial distribution of the roots and consequently the structural features of the cracks.

**Figure 3:**
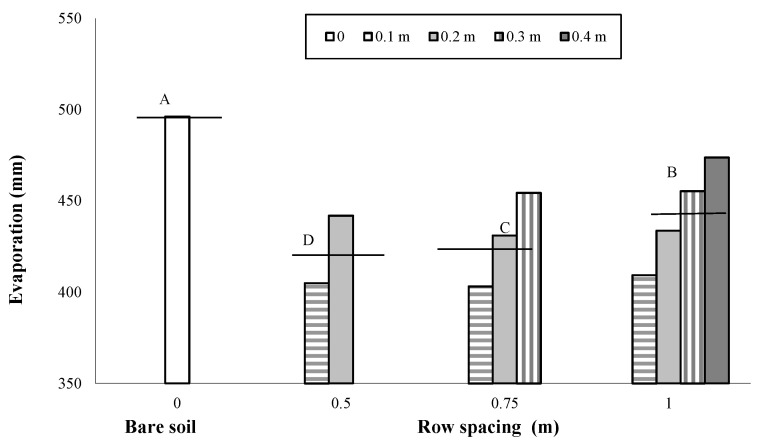
Evaporation measured from evaporation pans placed on the bare and cultivated soils as influenced by the change in row spacing (0 m vs. 0.5, 0.75 and 1 m) and in the distance from the row (0.1, 0.2, 0.3 and 0.4 m). The horizontal lines over the bars represent the mean values of the groups. The group means with the same letter are not significantly different (SNK test at *p* = 0.01).

As previously reported, a total amount of rainfall of 343 mm was recorded during the maize cropping cycle, with rain events quite evenly distributed over the season and with monthly values slightly higher than 60 mm in the period March–May and in July (Figure 1). Soil evaporation measured from the pan evaporimeters over the cropping cycle was higher from the bare soil (496 mm) than the soil cropped with maize (432 mm). In addition, in the cropped soil, the water loss by evaporation decreased with the decrease in row spacing and with the distance from the row due to the effect of crop shading (Figure 3). Specifically, decreasing the row spacing from 1 m to 0.75 and 0.50 m, the water losses through evaporation decreased by 3% and 5%, respectively (Figure 3).

**Figure 4:**
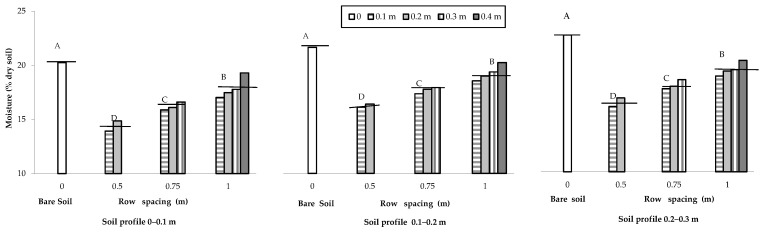
Moisture content of the bare and cultivated soils as influenced by the change in row spacing (0 m vs. 0.5, 0.75 and 1 m), along the soil depth and distance from the row (0.1, 0.2, 0.3 and 0.4 m). The horizontal lines over the bars represent the mean values of the groups. The group means with the same letter within each soil profile are not significantly different (SNK test at *p* = 0.01).

The soil water content measured close to the end of the maize cropping cycle was affected by plant transpiration. The soil water content was on average greater in the bare soil (21.24 g 100 g^−1^ of the dry weight) in comparison to the cropped soil (17.12 g 100 g^−1^ of the dry weight), where increased with the increase in the distance from the row and with the soil profile from the shallower (0–0.1 m) to the deeper (0.2–0.3 m) layers (Figure 4).

### 2.4. Root Density and Spatial Distribution

**Figure 5:**
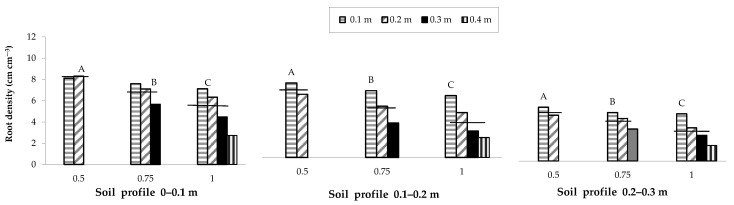
Root density of the maize crop as influenced by variation in row spacing (0.5, 0.75 and 1 m), soil profile (0–0.1, 0.1–0.2 and 0.2–0.3 m) and distance from the row (0.1, 0.2, 0.3 and 0.4 m). The horizontal lines over the bars represent the mean values of the groups. The group means with the same letter within each soil profile are not significantly different (SNK test at *p* = 0.01).

Overall, a lower soil water content was observed in the areas with greater root densities. In the same way, for the three plant densities investigated, root density decreased on average with the increase in the distance from the row and along the soil profile moving from the shallowest layer (0–0.1 m) to the deepest soil layer (0.2–0.3 m). Specifically, root density along the inter-row in the most superficial layer (0–0.1 m), which is the area with the greater root presence, decreased with the increase in row spacing; a row spacing of 0.5 m produced average decreases of 9.5 and 15.6% compared to spacings of 0.75 and 1 m, respectively (Figure 5).

**Figure 6:**
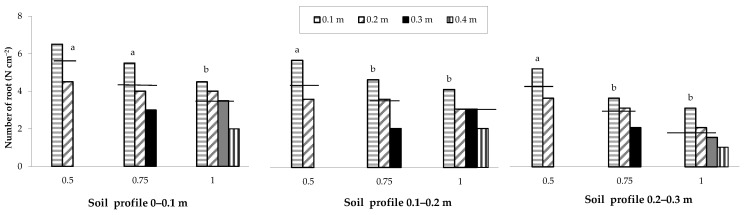
Spatial distribution of the maize root number as influenced by the change in row spacing (0.5, 0.75 and 1 m), soil profile and distance from the row (0.1, 0.2, 0.3 and 0.4 m). The horizontal lines over the bars represent the mean values of the groups. The group means with the same letter within each soil profile are not significantly different (SNK test at *p* = 0.05).

The spatial distribution of the root number, recorded by means of in situ mapping, showed a behaviour similar to that of root density as a function of the row spacing (Figure 6).

### 2.5. Structural Features of Soil Cracks

The pluviometric regime and the pedologic characteristics (sandy clay loam soil) recorded in the experimental site, as well as the presence of the crop grown under three different row spacings, affected the soil water content, the root spatial distribution and, consequently, the formation and development of desiccation cracks. 

All the parameters defining the crack features and network (length, width, depth and volume) showed an inverse behaviour with respect to the soil water content but a direct relationship with the root density. At the end of the maize growing season, after the drying cycle (35% of the available water), the soil cracks observed in the cultivated soil were parallel to the rows and characterized by width, length and depth greater than those observed on the bare soil. Indeed, in the non-cropped plots, soil cracks were smaller in size and showed an isotropic behaviour.

### 2.6. Crack Length, Width, Depth and Total Surface Area

Although the crack length is a soil feature not widely studied, its assessment can be useful to understand and model the cracking process and its intensity [24]. The total crack length (0.60 m m^−2^) was greater in the soil cropped with maize with a row spacing of 0.5 m, and significantly decreased with the increase in the row spacing; the smallest length was observed in the bare soil (0.17 m m^−2^) (Table 2).

The greatest crack width (0.08 m) was found with the narrower row spacing (0.5 m) which decreased with the increase in the spacing, being almost halved with a row spacing of 1 m (Table 2).

Changes in crack depth showed a similar behaviour to the one observed for crack width and length: the greater average depth value (0.31 m) was observed when the distance between the rows was smaller (Table 2). Crack depth and width features were significantly correlated as also derived by Equation 2.

The maximum average value of the crack surface (0.11 m^2^ m^−2^) was recorded under the narrower row spacing (0.5 m) and then decreased with the increase in the row spacing. A significantly lower value was recorded in the bare soil (0.03 m^2^ m^−2^) (Table 2).

### 2.7. Total Volume of Soil Cracks

**Figure 7:**
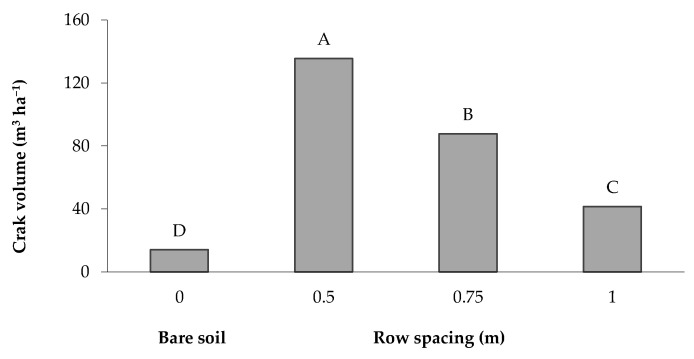
Volume of desiccation cracks in the bare soil and maize-cultivated soil under different row spacings. The bars with the same letter are not significantly different (SNK test at *p* = 0.01).

The total crack volume per surface unit represents a suitable indicator for characterizing the crack system and crack features, since it includes and summarizes the information brought by three dimensions (length, width and depth) of the soil cracks [25]. The greatest volume (135.65 m^3^ ha^−1^) was recorded in the soil cropped with a row spacing of 0.5 m and then decreased with the increase in the distance between the rows; the lowest value (14.05 m^3^ ha^−1^) was recorded in the bare soil (Figure 7). The crack volume, expressed as a percentage of the total soil volume in the 0–0.40 m layer, reached values greater than 1.4, 0.9 and 0.4% when maize was grown with a row spacing of 0.5, 0.75 and 1 m, respectively, whereas it was only 0.2% in the bare soil.

**Figure 8:**
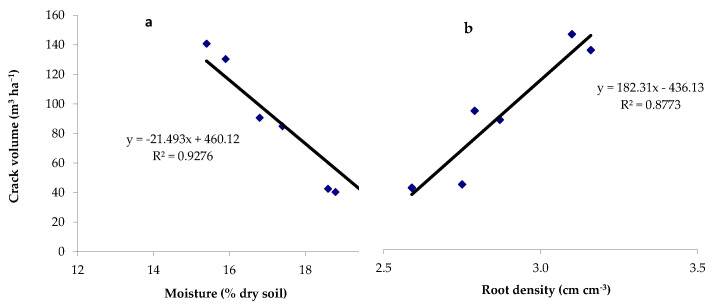
Linear regressions between the crack volume and (**a**) the gravimetric soil moisture content (*p* value = 0.0001) and (**b**) the root density (*p* value = 0.0059).

The evaporating surface notably increased when considering the area along the trapezoidal profile of the cracks. As reported in Figure 8, the crack volume showed a significant inverse relationship as a function of soil moisture content (*p* = 0.0001); a direct relationship was instead observed with root density (*p* = 0.0059).

## 3. Discussion

Over the recent decades, the improvement in cropping techniques and the introduction of hybrid varieties have led to a remarkable increase in maize yields [26]. This increase in crop yield of modern hybrid varieties can be seen as the result of maximized yield per plant under nonstress growing conditions associated with an improved tolerance to abiotic and biotic stresses [27]. Among the stress factors, modern hybrids are characterized by tolerance to “crowding” or plant density stress, which is essential for fully exploiting the yield per unit area [28,29]. It is well recognised that plant density represents an important stress factor, since a strong competition between different plant species or between different maize hybrids can markedly affect individual plant responses [30]. As a result of the improved stress tolerance of maize hybrids, greater plant densities and lower row spacings can be adopted, thus allowing a better exploitation of available resources (nutrients, water and light) [1,31]. A plant density up to 7 or even 10 plants per m^2^ has been reported to provide the optimum performance [32]. 

The highest kernel yield (16.57 Mg ha^−1^) was obtained with the maize crop grown with the highest planting density (6 plants m^−2^), with a row spacing of 0.5 m. A yield decrease of 8.09% was observed for the crop grown with a planting density of 4 plants m^−2^ (row spacing of 0.75 m). The lowest yield response, with a decrease of 18.24% in comparison to the highest yield value, was observed for the maize crop grown with the lowest planting density of 3 plants m^−2^ (row spacing of 1 m).

The greater plant density increases the competition among plants not only for water and nutrients but also for solar radiation and other growth factors. This strongly affects plant responses by causing a greater plant height, a lower stem robustness thus inducing a greater susceptivity to plant lodging, and a greater height of ear emergence. On the contrary, a lower plant density induces a lower competition of the plants and an increase in photosynthetic activity with a consequent better kernel development and a significant increase of the thousand seed and hectolitre weight. In particular, the hectolitre weight (or test weight), which is directly correlated to the thousand seed weight, is used as a general indicator of the overall yield quality, and a rough measure of endosperm hardness, kernel type and nutritive value of maize since these properties are related to dry-milling performance, post-harvest resistance to insects and starch digestibility of the maize [33].

The different planting strategies compared in this study significantly affected not only maize yield responses but also plant growth, nutritional status and root growth. A lower soil water content was observed in the areas characterized by greater root density; in addition, root density decreased along the inter-row and to the increase of the row spacing. Root density also decreased along the soil profile from the shallowest to the deepest layer. Similar findings were reported in other studies with a reduction of the root density with an increase in row spacing [17,34]. A similar behaviour was also observed for the root spatial distribution as a function of the row spacing.

In recent decades, an increase in the global temperature, together with a gradual decrease in atmospheric precipitation associated with its uneven distribution over the seasons and year, are causing the development or intensification of drought events [35,36,37]. For these reasons, in the future, a progressive reduction of the soil water availability for plant growth is expected [38,39]. This lower soil water content in arid and semi-arid environments will also result in the formation of soil desiccation cracks into the aerated unsaturated layer, particularly in fine-textured soils [40,41]. 

Changes in hydraulic conductivity can occur in clay soils during drying and wetting cycles with intensity varying as a function of the degree of soil plasticity. Soil desiccation cracks tend to develop during dry periods, whereas during rainfall events water can fill the cracks which is then slowly absorbed by the soil. Under water absorption, the cracks tend to disappear and the hydraulic conductivity decreases [42]. This behaviour is fundamental to ensure a greater exploitation capability of rainfall water and the building of a soil water reserve, which is crucial in soils characterized by low permeability.

The pedoclimatic conditions recorded in the experimental site, and the presence of the crop grown using different row spacings, affected the soil water content, the root spatial distribution and, consequently, the formation and development of desiccation cracks. An in-depth analysis of the crack features (length, width, depth and then total surface and volume) may provide important information. Previous studies have highlighted that variation in crack dimensions may depend on several factors such as changes in the pore size distribution, structural conditions, soil water content and structural stability of the soil aggregates [39]. The results of a previous study showed that crack width was affected by the duration of the drying period and crack depth was inversely related to the soil moisture content [43]. Different from the findings reported by Johnson [44], in our study the largest crack width was found with the narrower row spacing (0.5 m); crack width decreased with the increase in spacing resulting in a value being almost halved with a row spacing of 1 m. The narrowest cracks were instead observed in the bare soil, in agreement with the results found by Dasog and Shashidhara [45]. In addition, cracks depth and width features were significantly correlated as was also reported by other authors [46].

The crack surface describes the interface between soil and air, and therefore an increase in this feature enhances the water loss from the soil profile by evapotranspiration [25]. In our study, the greatest crack surface was recorded with the narrower row spacing (0.5 m) which decreased with the increase in row spacing; the lowest value was recorded from the bare soil.

Di Tommaso et al. [47] observed that the crop influenced the structural features of desiccation cracks with particular regard to the crack surface. On the cropped soil, a lower number of cracks with greater sizes than those observed on the bare soil was recorded. In clay soils with a high degree of plasticity at the end of the wheat cropping cycle, Ventrella et al. [48] observed the formation of many deep cracks that remained stable up to the execution of the main tillage. These cracks resulted in considerable water losses due to the increased soil evaporating surface and deep percolation [49]. In another study, the contact surface between the soil and the atmosphere reached values 4.5 times greater than that recorded in the same soil conditions when the soil was previously hoed [18].

Negative effects of desiccation cracks on agricultural fields have been reported [49] and are related to low nutrient retention, high evaporation rate and irregular seed germination and emergence [50]. As reported by Bordoloi et al. [51], different factors are involved in the formation and propagation of surface desiccation cracks, such as unsaturated soil mechanics (e.g., suction–moisture dynamics, initial compaction state), atmospheric conditions and vegetation characteristics. In particular, vegetation has been reported to induce highly contrasting effects in the development and expansion of desiccation cracks due to the interactions in the soil–water–plant–atmosphere continuum [51]. Plants modify the soil matric potential through root water uptake [52] causing soil desiccation and crack formation; on the other hand, plant roots may limit soil shrinkage thus minimizing crack expansion and evaporation from the cracks [51,53]. Plant roots can also proliferate inside cracks [54], further increasing crack width and depth.

There are few studies that relate cracking with variations in plant properties such as plant species, plant age, plant density and their spatial distribution [19,52]. Yang [55] reported that improper planting density or excessive productivity can cause denser cracks in soil. Planting strategy affects the initiation and the development of soil cracking and the cracking geometry, due to differences in transpiration and root spatial variability [51]. Other studies reported that the cracks would become wider when the inter-row spacing increased [44]. On the contrary, Cucci et al. [17] investigated the spatial distribution of soil cracks in two clay soils grown with sunflowers with different row spacings, and observed that the crack volume at a row spacing of 0.4 m was almost 8 times higher than that in the bare soil and 2.5 times higher than with a row spacing of 0.8 m.

In the present study, a similar trend in the soil cracking formation and features was observed. In particular, the greatest crack volume (135.65 m^3^ ha^−1^) was observed with a row spacing of 0.5 m, which then decreased with the increase in row spacing with values of 87.80 and 41.50 m^3^ ha^−1^ for the distances of 0.75 and 1 m, respectively. The minimum value of 14.05 m^3^ ha^−1^ was observed in the bare soil. The evaporating surface also notably increased in regard to the area along the crack profile and the increase of the crack volume during the soil drying cycle was inversely related with the soil moisture content. A linear relationship of the cracking parameters with the gravimetric soil moisture was also reported in other studies [21].

Our results showed that in these specific pedoclimatic conditions, the greatest crack volume was correlated with the highest maize productivity, in agreement with other studies [55]. A greater productivity results in a greater photosynthetic activity that would further increase the tensile forces in the root zone [56].

## 4. Materials and Methods

The research was carried out at the experimental farm “Martucci” of the University of Bari located in Valenzano (Southern Italy, 41°02′38″ N latitude, 16°90′66″ E longitude, 112 m above sea level), on a sandy clay loam red soil, a Chromic Luvisol type of soil according to the World Reference Base [57]. The main soil characteristics, measured from soil samples collected on the 0–0.4 m profile, are reported in Table 3. Physical and chemical analyses were performed according to the official methods [58].

The field experiment compared bare soil and soil cropped with maize (*Zea mais* L.) using three plant densities (6, 4 and 3 plants m^−2^) obtained by keeping the number of plants in a row (3 plants m^−1^) constant and varying the distance between the rows (0.5–0.75–1.0 m). The maize hybrid used was “P1547, Pioneer”, FAO class 600, with a medium-late cycle.

The treatments were arranged into a Latin square experimental design (LSD) with a plot area of 25 m^2^. The experimental area was previously cropped with artichoke (*Cynara cardunculus scolymus* L. Hayek).

Before crop sowing, which was performed in the first week of May 2014, fertilizers were applied at a rate of 100, 120 and 120 kg ha^−1^, of N, P_2_O_5_ and K_2_O, respectively; afterwards, 150 kg ha^−1^ of N was applied in top-dressing.

Weed control was carried out in post-emergence, at the phenological stage of 5th leaf, by applying a mixture of Nicosulfuron and Rimsulfuron (chemical family of sulphonylurea herbicides) at a rate of 90 g ha^−1^. Irrigation management consisted of returning to the entire soil mass the field water capacity at a depletion of 50% of the available water determined by the evaporation–transpiration method. Irrigation started at the beginning of stem elongation and a total water depth of 96 mm was applied.

All the other cultivation techniques were carried out according to the common techniques practiced in the area under study.

During the maize cropping cycle, the following parameters were measured: the evaporation from pan evaporimeters (located along the inter-row with increments of 0.1 m from the row) and the chlorophyll content using a chlorophyll meter (SPAD). In addition, at the end of the cropping cycle, the soil water content (using the thermo-gravimetric method), the distribution and features of soil cracks and the root distribution and density were measured. Finally, at harvesting, performed in the last week of September, the following yield response variables were measured: plant height, number of ears m^−2^, aboveground (shoot) dry biomass, kernel yield, thousand seed weight and hectolitre kernel weight. During the whole maize cropping cycle, air temperatures and rainfall were recorded from a meteorological station located in the experimental farm.

### 4.1. Measure of Crack Parameters

In each plot, four square areas with a side length of 1 m were identified. Within each square, the apparent lengths of the cracks on the soil surface were measured using a flexible meter inserted into the cracks [45].

The depth and the average width of the soil cracks were assessed based on measurements performed in different points chosen randomly along the cracks in each square.

At least one observation was done for each 0.5 m segment. The depth was measured using a steel reinforcing bar with a diameter of 2 mm inserted until there was resistance to further penetration. In the same position, the width was measured using a calliper at 1 cm below the soil surface [24]. The depth of 1 cm was chosen to avoid an overestimation of the crack width caused by surface dispersion.

The total volume (V, m^3^ per m^2^ of field surface area) and the surface (SA, m^2^) of the cracks were measured in the same sampling area of 1 m^2^ and computed assuming the section of each crack was an isosceles triangle and using the formula given in Dasog et al. [24] and Bandyopadhyay et al. [25]:

V = ∑ 0.5(1)

SA = ∑ *Cl*; C = [(0.5 *w*)^2^ + *d*^2^] ^½^ (2)

where *w* is the average crack width, *l* is the crack length, *d* is the average crack depth, and *C* is the parameter based on *w* and *d*.

### 4.2. Root Distribution

To evaluate the root distribution, starting from the centre of the inter-row of the sampling area, a trench was dug at 0.40 m and with progressive increments of 0.1 m both towards the row and along the profile. A paper-based map was then drawn following the methodology reported in Böhm [59]; soil samples were collected to quantify the root density according to the methodology reported in Greig Smith [60].

Soil samples (carrots), with a size of 7 cm (Ø) by 7.6 cm (h), were collected using a manual auger. The separation of the roots from the soil was performed in a laboratory by washing in order to also remove the organic residues; then, the roots were stored in an ethanol solution (10% *v*/*v*).

The root spatial distribution was assessed from maps in situ [59] identifying the roots on a vertical plane. The positions of the roots were recorded on transparent sheets arranged on the vertical observation plan, and the root distribution was assessed using the mean variance ratio, as reported in [60].

The Newman [61] method was applied for root length estimation. The method is based on the relationship between the number of intersections (*N*) originated from a sample of roots randomly arranged on a square mesh grid of area A and the total length of its reticle (*H*) according to the following equation (Equation (3)) that defines the total length of the roots (L):

(3) $$L=\frac{\pi NA}{2H}$$

T The root length density (RLD) was computed as the ratio of the root length to the soil volume unit. The root dry weight was instead not measured since, different from root length, it does not represent a suitable indicator of the plant adsorption capacity. This is shown by the fact that the thin roots, that are the most physiologically active, account for a very low percentage in weight of the whole root system.

### 4.3. Statistical Analysis

The data collected for all the investigated variables were analysed through an analysis of variance considering a Latin square design, using the GLM procedure of the SAS/STAT; differences between treatment means were assessed using the Student–Newman–Keuls (SNK) post hoc test. Linear regression analyses were also performed to describe how the volume of the soil cracks varied as a function of the soil water content and the root density.

## 5. Conclusions

The present study, carried out in Southern Italy on a sandy clay loam soil, investigated the effects of row spacing on maize growth and yield response, hydric relations in the soil–plant system continuum and desiccation crack features. The highest kernel yield of 16.57 Mg ha^−1^ was obtained using the highest planting density tested (6 plants m^−2^). The optimal row spacing of 0.5 m was able to provide a yield increase of 8.65% and 22.09% in comparison to the crops grown with a spacing of 0.75 and 1 m, respectively.

The different cropping systems affected the soil water content and, at the end of the growing season, the soil water content in the bare soil was on average 4% greater in comparison to the cropped soil. Soil water content was also affected by the different row spacings in the cropped soil, with a decrease in soil moisture observed with the decrease in the inter-row spacing. An inverse behaviour was observed between soil moisture and both the root density and soil crack size. Root density and root spatial distribution, recorded by means of in situ mapping, decreased due to the soil profile and the increase in the distance from the row.

The pluviometric regime occurred during the maize growing season (total rainfall of 343 mm) resulted in the formation of cracks of reduced sizes and with an isotropic behaviour in the bare soil, whereas in the cultivated soil, the cracks were parallel to the maize rows and increased in size with decreasing inter-row distance. The total volume of the soil cracks, a feature representing both the soil evaporating capacity and its ability to store water during rainy events, reached a value of 135.65 m^3^ ha^−1^ in the soil cropped with a row distance of 0.5 m, and was about ten times greater than the value observed for the bare soil and three times greater than the value observed with a row spacing of 1 m. Such a volume would allow a recharge of 14 mm in the case of intense rainy events on soil characterised by low permeability and would limit erosion phenomena in sloping soils with plant rows allocated in the direction orthogonal to the maximum slope lines.

In fine-grained soils, and especially clayey soils, that tend to show the formation of desiccation cracks because of swelling and shrinkage of clay minerals, agronomic management, with particular regard to row spacing, should be aimed to balance the needs of limiting crack formation and their size and ensuring optimal yield response in terms of both quantitative and qualitative results. In this study, the plant density at a row spacing of 0.75 m was able to combine optimal yield response in both quantitative and qualitative terms and lower crack dimensions, with particular regard to crack depth (21 cm vs. 31 cm), in comparison to the row spacing of 0.50 m.

## Figures and Tables

**Table 1:** Effect of row spacing on morphological, commercial and physiological parameters of maize.

Row Spacing	Plant Height	Ear Number	Shoot Dry Biomass	Kernel Yield *	Hectolitre Kernel Weight	Thousand Seed Weight
(m)	(cm)	(n m^−2^)	(Mg ha^−1^)	(Mg ha^−1^)	(kg hL^−1^)	(g)
0.50	296.5 a	9.75 a	29.81 a	16.57 a	70.95 c	311.55 c
0.75	282.3 b	6.50 b	26.16 b	15.23 b	71.53 b	340.00 b
1.00	269.0 c	4.75 c	23.20 c	13.58 c	74.25 a	352.07 a

* at 14% moisture content. For each variable considered, the values followed by the same letter are not significantly different, according to the SNK test at *p* = 0.05.

**Table 2:** Cracking properties in the bare soil and maize-cultivated soil under different row spacings.

Treatments	Length	Width	Depth	Area
	(m m^−2^)	(m)	(m)	(m^2^ m^−2^)
Bare soil	0.17 d	0.03 c	0.05 c	0.03 c
Row spacing 0.50	0.60 a	0.08 a	0.31 a	0.11 a
Row spacing 0.75	0.48 b	0.06 ab	0.21 b	0.09 ab
Row spacing 1.00	0.38 d	0.05 b	0.13 b	0.07 b

Different letters correspond to significantly different values according to SNK test at *p* = 0.05.

**Table 3:** Main physico-chemical and hydrologic proprieties of the soil.

Parameters	Units	Values
Total sand (2–0.02)		41.11
Silt (0.02–0.002)	(g 100 g^−1^)	31.02
Clay (<0.002)		27.87
Total Nitrogen (Kjeldahl method)	(g 1000 g^−1^)	1.48
Available phosphorus (Olsen method)	(mg kg^−1^)	18.00
Exchangeable potassium (BaCl_2_ method)	(mg kg^−1^)	260.00
Organic matter (Walkley Black method)	(g 100 g^−1^)	2.35
Total limestone (Dietrich–Fruhling calcimeter)	(g 100 g^−1^)	10.75
Ece	(dS m^−1^)	0.51
pH (pH in H_2_O)		7.75
CEC (BaCl_2_ method)	(meq 100 g^−1^)	30.40
Field capacity (field determination)	(g 100 g^−1^ d.m.)	27.19
Wilting point (−1.5 MPa)	(g 100 g^−1^ d.m.)	14.04
Bulk density	(kg dm^−3^ )	1.31

BaCl_2_, barium chloride; ECe, saturation extract electrical conductivity; CEC, cation exchange capacity.

## Data Availability

The data that support the findings of this study are available from the corresponding author upon reasonable request.
